# Immature Neurons and Radial Glia, But Not Astrocytes or Microglia, Are Altered in Adult Cntnap2 and Shank3 Mice, Models of Autism

**DOI:** 10.1523/ENEURO.0196-16.2016

**Published:** 2016-10-17

**Authors:** Elise C. Cope, Brandy A. Briones, Adam T. Brockett, Susana Martinez, Pierre-Antoine Vigneron, Maya Opendak, Samuel S.-H. Wang, Elizabeth Gould

**Affiliations:** 1 Princeton Neuroscience Institute, Princeton University, Princeton, New Jersey 08540; 2 Department of Psychology, Princeton University, Princeton, New Jersey 08544; 3 Department of Molecular Biology, Princeton University, Princeton, New Jersey 08544

**Keywords:** astrocytes, autism, microglia, neurogenesis, radial glia

## Abstract

Autism spectrum disorder (ASD) is often associated with cognitive deficits and excessive anxiety. Neuroimaging studies have shown atypical structure and neural connectivity in the hippocampus, medial prefrontal cortex (mPFC), and striatum, regions associated with cognitive function and anxiety regulation. Adult hippocampal neurogenesis is involved in many behaviors that are disrupted in ASD, including cognition, anxiety, and social behaviors. Additionally, glial cells, such as astrocytes and microglia, are important for modulating neural connectivity during development, and glial dysfunction has been hypothesized to be a key contributor to the development of ASD. Cells with astroglial characteristics are known to serve as progenitor cells in the developing and adult brain. Here, we examined adult neurogenesis in the hippocampus, as well as astroglia and microglia in the hippocampus, mPFC, and striatum of two models that display autism-like phenotypes, Cntnap2^−/−^ and Shank3^+/ΔC^ transgenic mice. We found a substantial decrease in the number of immature neurons and radial glial progenitor cells in the ventral hippocampus of both transgenic models compared with wild-type controls. No consistent differences were detected in the number or size of astrocytes or microglia in any other brain region examined. Future work is needed to explore the functional contribution of adult neurogenesis to autism-related behaviors as well as to temporally characterize glial plasticity as it is associated with ASD.

## Significance Statement

ASD is a heterogeneous developmental condition that is estimated to affect 1 in 68 children under 8 years of age in the United States. Postmortem studies suggest that both neurons and glia may be compromised in ASD, but few studies have explored neural and glial plasticity across relevant mouse models. We found a decrease in adult neurogenesis in the ventral hippocampus, a brain region important for anxiety and stress regulation, in two autism mouse models compared with wild-type controls. However, we found no differences in markers of glial dysregulation across autism mouse models in the hippocampus, medial prefrontal cortex, or striatum, suggesting that persistent abnormalities in glia in these brain regions are not necessary for the ASD behavioral phenotype.

## Introduction

ASD is characterized by deficits in social communication, repetitive behaviors, and/or restricted interests (American Psychiatric Association, 2013), and is often associated with cognitive deficits (Goh and Peterson, 2012) and increased prevalence of anxiety disorders (Vasa and Mazurek, 2015; Russell et al., 2016). Neuroimaging and postmortem studies of ASD individuals have identified an atypical structure in a variety of brain regions, including, but not limited to, the hippocampus, the medial prefrontal cortex (mPFC), and the striatum (Schumann et al., 2004; Carper and Courchesne, 2005; Langen et al., 2009), regions implicated in the regulation of cognition, anxiety, and stereotyped behavior. Additionally, postmortem studies have revealed greater numbers of dendritic spines (Hutsler and Zhang, 2010; Penzes et al., 2011), sites of excitatory synapses, when compared with controls, potentially due to abnormal synapse elimination during prenatal and postnatal development.

ASD is a highly heritable disorder with concordance rates as high as 90% between monozygotic twins and 30% between siblings (Rosenberg et al., 2009; Geschwind, 2011; Ozonoff et al., 2011). Furthermore, studies have identified hundreds of genes associated with the disorder, many of which encode for proteins important for synaptic structure and function (Geschwind, 2011; Parikshak et al., 2013). Transgenic mice based on rare mutations offer tractable models with which to study the development of ASD, test potential treatments, as well as study changes in cell function and behavior (Lázaro and Golshani, 2015). Knockout of Cntnap2, a gene encoding for a neuronal transmembrane protein that is thought to be important for the control of neuron–glia interactions, is associated with alterations in synaptic structure, neuronal network activity, and an autism-related phenotype in mice (Peñagarikano et al., 2011; Gdalyahu et al., 2015). Shank3 mutations are linked to the development of Phelan-McDermid Syndrome (22q13 deletion syndrome), a disorder that presents with many of the core symptoms of ASD as well as global developmental delays (Jiang and Ehlers, 2013). Shank3 encodes for a scaffolding protein that is involved in the maintenance of the postsynaptic density. Mice with partial or complete deletion of Shank3 have deficits in social interaction and cognition as well as alterations in synaptic physiology (Jiang and Ehlers, 2013; Kouser et al., 2013).

Several lines of evidence point to an important role for glial dysregulation in ASD. Prenatal maternal illness and stress, conditions that stimulate the maternal immune system and induce reactive gliosis (Zager et al., 2015), predispose offspring to develop ASD (Kinney et al., 2008; Atladóttir et al., 2010). Not only have postmortem studies shown that individuals with ASD have increased numbers of astrocytes and microglia in a variety of brain regions (Vargas et al., 2005; Morgan et al., 2010), but these studies have also revealed abnormalities in peripheral proinflammatory cytokine levels in individuals with ASD (Ashwood et al., 2011; Xu et al., 2015). While traditionally thought of as support cells, recent evidence has implicated a role for astrocytes in synapse formation (Christopherson et al., 2005; Kucukdereli et al., 2011) and elimination (Chung et al., 2013), as well as in synaptic plasticity (Henneberger et al., 2010). Microglia, the resident immune cells of the brain, are also known to sculpt neural circuits by engulfing weak synapses during development (Paolicelli et al., 2011; Schafer et al., 2012). Reactive microglia produce several inflammatory molecules that can directly impact synaptic plasticity by altering firing rates and degrading synapses (Kondo et al., 2011). Since both astrocytes and microglia are involved in shaping and modulating neural connectivity, their early disruption may contribute to abnormal brain development and an ASD phenotype.

In addition to glial plasticity, another form of plasticity, adult neurogenesis in the hippocampus, may also be altered in ASD. Reduced adult neurogenesis in the hippocampus has been reported in the BTBR mouse model of autism (Stephenson et al., 2011); however, no work has explored the possibility that this is a feature common to other ASD mouse models. Because adult neurogenesis has been linked to a variety of hippocampus-mediated behaviors, including learning and memory, anxiety regulation, as well as social behaviors (Opendak and Gould, 2015; Opendak et al., 2016), it is possible that alterations in adult neurogenesis may underlie part of the abnormal behavioral phenotype seen in autism.

To explore the possibility that genetic mouse models of ASD are associated with differences in neuronal and glial plasticity, we examined the number of new neurons and radial glia in the hippocampus, as well as the number, morphology, and reactivity of astrocytes and microglia in the hippocampus, mPFC, and striatum of Cntnap2^−/−^, Shank3^+/ΔC^, and wild-type (WT) male mice.

## Materials and Methods

### Animals

All animal procedures were performed in accordance with the Princeton University Institutional Animal Care and Use Committee regulations and conformed to the National Research Council Guide for the Care and Use of Laboratory Animals (2011). Adult male C57BL/6J (catalog #000664) and Cntnap2^−/−^ (catalog #017482) mice were obtained from The Jackson Laboratory. Shank3^+/ΔC^ (catalog #018398) mice were originally obtained from The Jackson Laboratory and bred at Princeton University using a heterozygote–heterozygote strategy. For all experiments, 5- to 6-month-old mice were used. Since there is an increased prevalence of ASD among males (Christensen et al., 2012), only male mice were used for these studies (*n* = 5-10/group). All mice were group housed in Optimice cages on a reverse 12 h light/dark cycle.

### Immunohistochemistry

Mice were deeply anesthetized with Euthasol and transcardially perfused with 4% paraformaldehyde (PFA) in PBS. Following perfusions, brains were postfixed for 48 h in 4% PFA followed by cryoprotection with sucrose for 48 h. 40-μm-thick coronal sections were cut from half brains using a Cryostat (Leica Biosystems). For immature neuron immunolabeling, free-floating sections throughout the hippocampus were rinsed in Tris-buffered saline (TBS) and then incubated with 0.5% Tween-20, 3% normal donkey serum, and goat anti-doublecortin (DCX; 1:100; Santa Cruz Biotechnology) in TBS at 4°C for 48 h. Sections were then washed and incubated with Alexa Fluor donkey anti-goat 568 (1:250; Invitrogen) for 1 h at room temperature.

Astrocyte and microglial immunolabeling were conducted separately on free-floating sections that were rinsed in PBS, incubated with 3% normal donkey serum, PBS with 0.1% Triton X-100, and rabbit anti-glial fibrillary acidic protein (GFAP; 1:500; Dako), a cytoskeletal astrocyte marker; rabbit anti-S100 (1:10,000, Dako), a Ca^2+^ binding protein that labels astrocyte cell bodies; or rabbit anti-ionized calcium-binding adapter molecule 1 (Iba1; 1:500; Wako), a microglial cell body marker; and rat anti-CD68 (1:200; Serotec), a microglial lysosomal marker. All tissue was incubated in primary antisera for 24 h at 4°C, and then rinsed and incubated with secondary antisera, consisting of either Alexa Fluor donkey anti-rabbit 488 (1:250; Invitrogen) or Alexa Fluor donkey anti-rabbit 568 (1:250; Invitrogen) for astrocyte analyses, or both Alexa Fluor donkey anti-rabbit 488 (1:250; Invitrogen) and Alexa Fluor donkey anti-rat 568 (1:250; Invitrogen) for microglial analyses. Sections were kept in the dark for 1 h, at which point they were rinsed, mounted onto Suprafrost slides, and coverslipped using glycerol in PBS (3:1).

### Cell density measurements

Slides were coded until completion of the data analysis. Since the dorsal and ventral regions of the hippocampus are considered functionally distinct (Fanselow and Dong, 2010), all measures in these regions were analyzed separately (dorsal to bregma, −0.94 to −3.16 mm; ventral to bregma, −3.16 to −3.88 mm; Franklin and Paxinos, 2008). For dorsal hippocampus, mPFC, and striatum, four to six sections were analyzed for cell densities. Since the anterior–posterior extent of the ventral hippocampus is relatively small compared with the dorsal hippocampus, fewer sections were analyzed for this subregion (two to three sections). Because the boundaries of the hippocampus were easy to define, quantitative analyses of cell densities (DCX, GFAP, S100, and Iba1) in this brain region were obtained from counts of the overall structure using Stereo Investigator software (Microbrightfield Bioscience). Briefly, the extent of the individual subregions [subgranular zone (SGZ), molecular layer (MOL), and stratum radiatum (RAD)] were outlined, and any positive cells within the delineated region were counted exhaustively. Densities were determined by dividing the total number of positive cells by the volume of the region outlined. Since GFAP^+^ cells in the subgranular zone with radial morphology represent radial-glial-like progenitor cells with stem cell properties (Lugert et al., 2010), GFAP^+^ cells exhibiting radial and horizontal morphologies were counted separately. Because the precise boundaries of the mPFC (layers 2/3), dorsolateral striatum (DLS), and dorsomedial striatum (DMS) are more difficult to determine, density measurements were made anterior to posterior from the center of each structure following mouse brain atlas coordinates (Franklin and Paxinos, 2008), taking care to avoid the edges, where cells from adjacent structures might be inadvertently included. Cells were counted exhaustively from 20 µm image stacks obtained by use of a Zeiss confocal microscope (LSM 700; lasers: argon 458/488, HeNe 568) followed by processing in ImageJ (NIH). Densities were determined by dividing the total number of positive cells by the volume of the region contained in the stack.

### Astroglia analyses

Astroglia morphology was analyzed using S100 for cell body area and GFAP for cell domain area. Cross-sectional cell body area measurements of 50 S100^+^ cells (10–25 cells per section) were randomly selected per animal per brain region (hippocampus, mPFC, and striatum) from a minimum of two sections per brain region per animal. For GFAP domain area analyses, 50 cells per region per animal (10-25 cells per section for each brain region) were randomly selected from a minimum of two sections per brain region per animal and traced by closely following the contours of the distally stained processes using ImageJ (NIH; Oberheim et al., 2009). The *z*-stack images for S100 and GFAP were obtained using a 20× objective with 2× zoom and a 3 µm *z*-step. Because GFAP^+^ cells in the SGZ are either horizontal or radial-glial-like precursor cells, the GFAP domain area was not examined in this subregion.

### Microglial analyses

Iba1^+^ microglial cells were analyzed for the number of primary processes, cell body area, and activation status. Cross-sectional cell body area measurements of 10 randomly selected Iba1^+^ cells (two to five cells per section for each brain region) per animal were obtained from 20 µm image *z*-stacks using ImageJ (NIH). The *z*-stack images for Iba1^+^ were obtained with a 63× objective with a 0.7× zoom and a 0.56 µm *z*-step. In order to fully analyze the microglia processes, only Iba1-labeled cells in which the cell body was located toward the middle portion of the *z*-plane were selected for imaging.

Iba1^+^ microglial cells colabeled with lysosomal marker CD68, a marker of microglial activation, were assessed using two methods. The first method involved obtaining area measurements of CD68 staining in Iba1^+^ cells using a confocal microscope and ImageJ software. The second method was adapted from Schafer et al. (2012) and involved manual counting of CD68-stained aggregates within Iba1^+^ microglial cells. For both methods, *z*-stack images were collected for 10 individual Iba1^+^ cells (two to five cells per section) from each brain region per animal using a 63× objective with a 0.7× zoom and a 0.56 µm *z*-step on a Zeiss confocal microscope (LSM 700). Maximum intensity *z*-projections of the image stacks were created using ImageJ (NIH). For the manual counting method, aggregates were counted from the cell body and the processes of each individual microglial cell.

### Statistics

Data collection and analyses were performed by an experimenter who was blind to the animal group. For all experiments, data were analyzed by one-way ANOVA with Bonferroni *post hoc* comparisons (Table 1), except data sets that violated assumptions of homogeneity of variance or normal distribution (determined by the use of Bartlett’s tests). Those datasets were analyzed using Kruskal–Wallis, followed by Dunn’s, tests. GraphPad Prism 6.0 (GraphPad Software) was used for all the statistical analyses and graph preparation.

**Table 1:** Statistical table

Figure	Description	Data Structure	HOV	Type of test	95% CI for WT	95% CI for Cntnap2^−/−^	95% CI for Shank3^+/Δ^
1B	DCX in the dorsal hippocampus	Normal	Yes	One-way ANOVA	14,077–17,780	9,654–17,352	11,360–18,927
1D	DCX in the ventral hippocampus	Normal	Yes	One-way ANOVA	9,513–17,496	4,572–11,025	6,619–9,370
2C	rGCs and hGCs in the dorsal hippocampus	Normal	Yes	One-way ANOVA	8,381–10,624 (rGCs) 15,576–17,671 (hGCs)	8,709–10,961 (rGCs) 14,914–16,793 (hGCs)	7,668–11,762 (rGCs) 15,604–19,204 (hGCs)
2F	rGCs and hGCs in the ventral hippocampus	Normal	Yes	One-way ANOVA	6,766–10,273 (rGCs) 16,066–19,354 (hGCs)	3,469–7,123 (rGCs) 18,018–20,874 (hGCs)	4,636–6,844 (rGCs) 17,658–21,043 (hGCs)
N/A	rGCs and DCX cells in the hippocampus	Normal	Yes	One-way ANOVA	0.51–0.71 (dorsal) 0.47–0.82 (ventral)	0.59–0.89 (dorsal) 0.57–0.98 (ventral)	0.51–0.82 (dorsal) 0.49–0.89 (ventral)
3B	GFAP density in the dorsal hippocampus	Non-normal (MOL)Normal (RAD)	No (MOL)Yes (RAD)	Kruskal–Wallis (MOLOne-way ANOVA (RAD)	30,863–35,020 (MOL)27,638–32,713 (RAD)	31,880–35,640 (MOL)27,138–31,130 (RAD)	26,868–39,752 (MOL)27,501–32,706 (RAD)
3C	GFAP domain area in the dorsal hippocampus	Normal	Yes	One-way ANOVA	795–1,063 (MOL)912–1,168 (RAD)	756–935 (MOL)869–1,202 (RAD)	731–870 (MOL)630–899 (RAD)
3E	GFAP density in the ventral hippocampus	Normal	Yes	One-way ANOVA	33,738–37,726 (MOL)25,884–30,244 (RAD)	36,894–40,994 (MOL)24,924–29,812 (RAD)	30,727–39,249 (MOL)23,393–30,019 (RAD)
3F	GFAP domain area in the ventral hippocampus	Normal	Yes	One-way ANOVA	749–911 (MOL)846–1,154 (RAD)	669–860 (MOL)825–1,346 (RAD)	635–860 (MOL)905–1,185 (RAD)
4B	S100 density in the dorsal hippocampus	Normal	Yes	One-way ANOVA	17,245–21,751 (MOL)15,107–18,016 (RAD)	14,940–17,997 (MOL)12,518–15,539 (RAD)	12,911–21,489 (MOL)11,923–18,519 (RAD)
4C	S100 morphology in the dorsal hippocampus	Normal	Yes	One-way ANOVA	51–64 (MOL)48–60 (RAD)	51–65 (MOL)51–60 (RAD)	44–63 (MOL)47–63 (RAD)
4E	S100 density in the ventral hippocampus	Normal	Yes	One-way ANOVA	19,035–25,859 (MOL)13,518–17,458 (RAD)	14,146–19,274 (MOL)11,222–13,369 (RAD)	15,336–23,375 (MOL)11,410–16,797 (RAD)
4F	S100 morphology in the ventral hippocampus	Normal	Yes	One-way ANOVA	47–59 (MOL)55–64 (RAD)	48–61 (MOL)58–64 (RAD)	42–57 (MOL)53–65 (RAD)
5B	Iba1 density in the dorsal hippocampus	Normal	Yes	One-way ANOVA	8,089–10,437 (MOL)8,091–9,830 (RAD)	8,800–10,325 (MOL)8,378–9,594 (RAD)	8,742–10,086 (MOL)8,160–9,113 (RAD)
5C	Iba1 cell body area in the dorsal hippocampus	Normal	Yes	One-way ANOVA	44–47 (MOL)41–43 (SGZ)41–45 (RAD)	44–47 (MOL)42–44 (SGZ)42–45 (RAD)	43–48 (MOL)41–45 (SGZ)42–46 (RAD)
N/A	Iba1 processes in the dorsal hippocampus	Normal	Yes	One-way ANOVA	5.38–6.49 (MOL)4.58–5.40 (SGZ)5.58–7.46 (RAD)	5.96–6.82 (MOL)5.11–5.78 (SGZ)5.05–7.37 (RAD)	5.56–6.50 (MOL)5.16–6.36 (SGZ)6.53–7.87 (RAD)
5E	Iba1 density in the ventral hippocampus	Normal	Yes	One-way ANOVA	8,799–11,297 (MOL)7,208–8,912 (RAD)	7,995–9,825 (MOL)7,500–9,681 (RAD)	8,597–10,955 (MOL)7,279–8,814 (RAD)
5F	Iba1 cell body area in the ventral hippocampus	Normal (MOL, SGZ)Non-normal (RAD)	Yes (MOL, SGZ)No (RAD)	One-way ANOVA (MOL, SGZ)Kruskal–Wallis (RAD)	43–45 (MOL)40–46 (SGZ)40–48 (RAD)	43–46 (MOL)39–45 (SGZ)40–46(RAD)	43–46 (MOL)43–47 (SGZ)43–46 (RAD)
N/A	Iba1 processes in the ventral hippocampus	Normal	Yes	One-way ANOVA	5.69–7.19 (MOL)4.58–5.78 (SGZ)5.37–6.95 (RAD)	6.68–7.78 (MOL)4.83–5.83 (SGZ)5.34–7.26 (RAD)	5.32–7.05 (MOL)4.62–5.44 (SGZ)5.88–7.66 (RAD)
6B	CD68 percent area in the dorsal hippocampus	Normal	Yes	One-way ANOVA	25–52 (MOL)30–60 (SGZ)44–71 (RAD)	37–64 (MOL)23–51 (SGZ)46–73 (RAD)	36–60 (MOL)29–70 (SGZ)42–76 (RAD)
6D	CD68 percent area in the ventral hippocampus	Normal (MOL, SGZ)Non-normal (RAD)	Yes (MOL, SGZ)No (RAD)	One-way ANOVA (MOL, SGZ)Kruskal–Wallis (RAD)	34–57 (MOL)35–65 (SGZ)43–68 (RAD)	33–63 (MOL)30–54 (SGZ)70–75 (RAD)	36–60 (MOL)25–66 (SGZ)48–77 (RAD)
N/A	CD68 aggregates in the dorsal hippocampus	Normal	Yes	One-way ANOVA	1.0–1.7 (MOL)2.4–3.3 (SGZ)1.1–1.4 (RAD)	1.4–2.6 (MOL)2.6–3.2 (SGZ)1.1–1.8 (RAD)	1.3–2.4 (MOL)1.6–2.9 (SGZ)1.1–2.1 (RAD)
N/A	CD68 aggregates in the ventral hippocampus	Normal (MOL, SGZ)Non-normal (RAD)	Yes (MOL, SGZ)No (RAD)	One-way ANOVA (MOL, SGZ)Kruskal–Wallis (RAD)	0.73–1.2 (MOL)2.7–3.9 (SGZ)1.0–1.7 (RAD)	0.89–1.7 (MOL)2.8–3.6 (SGZ)1.2–1.8 (RAD)	0.85–2.1 (MOL)3.5–4.3 (SGZ)1.0–2.1 (RAD)
7B	GFAP density in the mPFC	Normal	Yes	One-way ANOVA	14,284–19,637	15,287–21,324	11,541–21,324
7C	GFAP domain area in the mPFC	Normal	Yes	One-way ANOVA	672–894	404–818	558–876
8B	S100 density in the mPFC	Normal	Yes	One-way ANOVA	37,563–42,408	34,360–41,972	33,026–44,822
8C	S100 cell body area in the mPFC	Normal	Yes	One-way ANOVA	57–75	54–65	59–76
9B	Iba1 density in the mPFC	Normal	Yes	One-way ANOVA	16,458–20,885	17,605–19,476	14,107–22,088
9C	Iba1 cell body area in the mPFC	Normal	Yes	One-way ANOVA	49–59	52–61	53–61
N/A	Iba1 processes in the mPFC	Normal	Yes	One-way ANOVA	4.27–4.97	4.55–5.11	3.99–5.13
10B	CD68 percent area in the mPFC	Normal	Yes	One-way ANOVA	31–42	28–39	31–53
N/A	CD68 aggregates in the mPFC	Normal	Yes	One-way ANOVA	1.9–3.0	2.0–3.2	1.5–2.6
11B	GFAP density in the DLS	Normal	Yes	One-way ANOVA	4,576–9,999	2,227–10,600	3,141–13,608
11C	GFAP domain area in the DLS	Normal	Yes	One-way ANOVA	894.1–1,167	893.7–1,281	731.1–1,238
11E	GFAP density in the DMS	Normal	Yes	One-way ANOVA	5,431–12,016	4,384–13,134	4,747–15,096
11F	GFAP domain area in the DMS	Normal	Yes	One-way ANOVA	794.6–1,085	791.2–1,137	623.3–1,144
12B	S100 density in the DLS	Normal	Yes	One-way ANOVA	14,376–20,394	15,624–24,349	8,112–18,553
12C	S100 morphology in the DLS	Normal	Yes	One-way ANOVA	39.37–46.03	41.13–46.35	39.71–46.62
12E	S100 density in the DMS	Normal	Yes	One-way ANOVA	21,978–29,554	20,744–29,426	12,672–23,759
12F	S100 morphology in the DMS	Normal	Yes	One-way ANOVA	42.78–50.88	40.93–49.1	38.35–47.89
13B	Iba1 density in the DLS	Non-normal	No	Kruskal–Wallis	8,376–13,633	8,812–11,570	7,593–9,335
13C	Iba1 cell body area in the DLS	Normal	Yes	One-way ANOVA	47.11–52.55	46.7–53.66	44.12–49.94
N/A	Iba1 processes in the DLS	Normal	Yes	One-way ANOVA	5.92–7.52	5.89–7.23	5.80–6.97

13E	Iba1 density in the DMS	Non-normal	No	Kruskal–Wallis	7,618–13,277	8,374–10,970	6,749–9,419
13F	Iba1 cell body area in the DMS	Normal	Yes	One-way ANOVA	48.51–55.59	51.05–57.81	45.53–51.25
N/A	Iba1 processes in the DMS	Normal	Yes	One-way ANOVA	5.25–5.85	5.97–7.45	4.75–6.16
14B	CD68 percent area in the DLS	Non-normal	No	Kruskal–Wallis	62.06-83.87	74.36-80.24	59.26-84.94
14D	CD68 percent area in the DMS	Non-normal	No	Kruskal–Wallis	61.11-82.62	75.6-79.02	52.09-84.29
N/A	CD68 aggregates in the DLS	Non-normal	No	Kruskal–Wallis	2.43-4.67	3.21-4.11	2.1-3.73
N/A	CD68 aggregates in the DMS	Normal	Yes	One-way ANOVA	1.84-3.32	2.14-3.86	1.77-2.95

hGC, Horizontal glial cell; rGC, radial glial cell.

## Results

### Immature neurons in the hippocampus

**Figure 1.**
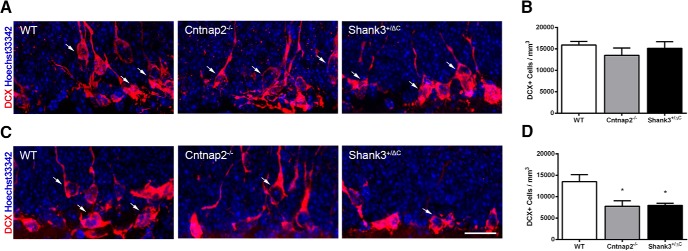
Cntnap2^−/−^ and Shank3^+/ΔC^ mice have reduced numbers of immature neurons in the ventral dentate gyrus of the hippocampus. ***A***, ***C***, Representative images of sections immunolabeled with the immature neuronal marker DCX (red) and counterstained with Hoechst 33342 (blue) from WT, Cntnap2^−/−^, and Shank3^+/ΔC^ mice in the dorsal (***A***) and ventral (***C***) dentate gyrus. Scale bar, 20 µm, applies to all frames. Arrows point to DCX^+^ cells. ***B***, ***D***, Quantification of the number of new neurons in the dorsal (***B***) and ventral (***D***) dentate gyrus. Error bars represent the SEM. *p < 0.05.

No differences were detected in the number of DCX^+^ cells in the dorsal dentate gyrus across mouse models (*F*_(2,24)_ = 0.87, *p* = 0.43; Fig. 1*A*,*B*). However, there was a significant difference in the ventral dentate gyrus (*F*_(2,15)_ = 6.18, *p* = 0.01; Fig. 1*C*,*D*). *Post hoc* analyses showed a statistically significant decrease, such that there was a >40% reduction in the mean densities of immature DCX^+^ neurons in the ventral dentate gyrus in both mouse models of autism compared to wild-type mice (WT vs Cntnap2^−/−^, *p* = 0.02; WT vs Shank3^+/ΔC^, *p* = 0.02).

### Astroglia in the hippocampus

**Figure 2.**
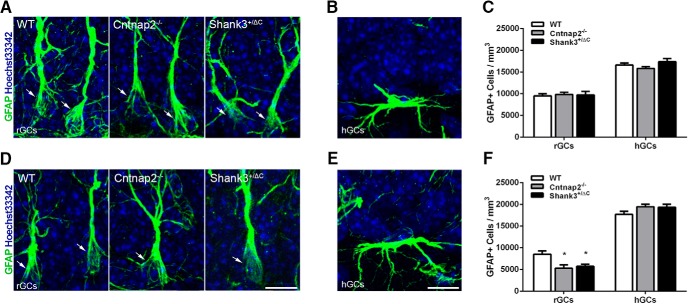
Cntnap2^−/−^ and Shank3^+/ΔC^ mice have reduced numbers of GFAP-labeled radial glial progenitor cells in the ventral dentate gyrus of the hippocampus ***A***, ***D***, Representative images of radial glial cells (rGCs) immunolabeled with astrocyte marker GFAP (green) and counterstained with Hoechst 33342 (blue) from the dorsal (***A***) and ventral (***D***) SGZ. Arrows point to GFAP^+^ radial glial cells. Scale bar, 10 µm, applies to all frames. ***B***, ***E***, Representative images of horizontal glial cells (hGCs) from the dorsal (***B***) and ventral (***E***) SGZ. Scale bar, 10 µm, applies to all frames. ***C***, ***F***, Quantification of the density of GFAP-labeled cells with radial glial morphology and horizontal morphology in the dorsal (***C***) and ventral (***F***) SGZ. Error bars represent the SEM. *p < 0.05.

**Figure 3.**
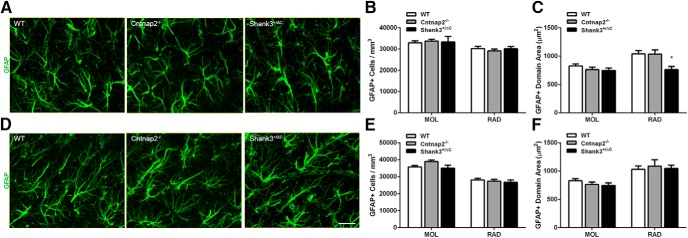
No consistent differences were observed in GFAP-labeled astrocyte density or domain size across mouse autism models in the hippocampus. ***A***, ***D***, Representative images of sections immunolabeled with astrocyte marker GFAP (green) from WT, Cntnap2^−/−^, and Shank3^+/ΔC^ mice in the dorsal (***A***) and ventral (***D***) MOL of the dentate gyrus. Scale bar, 20 µm, applies to all frames. ***B***, ***E***, Quantification of the density of GFAP-labeled astrocytes in the dorsal (***B***) and ventral (***E***) hippocampus. ***C***, ***F***, Quantification of GFAP cell domain area in the dorsal (***C***) and ventral (***F***) hippocampus. Error bars represent the SEM. *p < 0.05.

Quantitative analyses of the number of GFAP-stained astrocytes in the dorsal dentate gyrus revealed no significant differences between transgenic and wild-type mice in the SGZ (radial morphology: *F*_(2,23)_ = 0.09, *p* = 0.91; horizontal morphology: *F*_(2,23)_ = 2.03, *p* = 0.15; Fig. 2*A–C*), the MOL of the dentate gyrus (H = 0.98, 2 df, *p* = 0.61; Fig. 3*A*,*B*), or the RAD of the CA1 region (*F*_(2,24)_ = 0.34, *p* = 0.72; Fig. 3*A*,*B*). Within the ventral SGZ, an overall significant effect in the number of GFAP^+^ cells with radial glial morphology, astroglial cells known to serve as progenitors for new neurons in the adult dentate gyrus, was observed (*F*_(2,22)_ = 6.34, *p* = 0.007; Fig. 2*D*,*F*). *Post hoc* analyses showed a significant decrease in density across both transgenic mouse models compared with wild-type controls (WT vs Cntnap2^−/−^, *p* = 0.007; WT vs Shank3^+/ΔC^, *p* = 0.03). By contrast, no difference was observed in this same region in the number of GFAP^+^ cells with horizontal morphology (*F*_(2,22)_ = 2.15, *p* = 0.14; Fig. 2*E*,*F*). Examination of the proportion of the number of radial glia to the number of DCX^+^ cells revealed no differences in either the dorsal SGZ (*F*_(2,23)_ = 1.37, *p* = 0.27) or the ventral SGZ (*F*_(2,13)_ = 0.78, *p* = 0.48). There was an overall statistical significance in the density of GFAP^+^ cells in the ventral MOL (*F*_(2,24)_ = 3.55, *p* = 0.04; Fig. 3*D*,*E*), but *post hoc* analysis showed no differences between groups (WT vs Cntnap2^−/−^, *p* = 0.09; WT vs Shank3^+/ΔC^, *p* > 0.99). No differences in GFAP^+^ cell density were detected in the ventral RAD (*F*_(2,24)_ = 0.35, *p* = 0.71; Fig. 3*D*,*E*). A significant decrease in the domain size (the maximum extent of the GFAP-stained processes) of individual GFAP^+^ astrocytes was observed in the dorsal RAD (*F*_(2,24)_ = 5.13, *p* = 0.01) in Shank3^+/ΔC^ mice compared with wild-type mice (*p* = 0.02), but this effect was not observed in Cntnap2^−/−^ mice (*p* > 0.99), which were virtually identical to wild-type mice on this measure. Moreover, the decrease in GFAP^+^ astrocyte domain size was not observed in the dorsal MOL (*F*_(2,24)_ = 1.79, *p* = 0.19; Fig. 3*C*) or in any of the subregions examined in the ventral hippocampus (MOL: *F*_(2,24)_ = 1.12, *p* = 0.34; RAD: *F*_(2,24)_ = 0.25, *p* = 0.78; Fig. 3*F*).

**Figure 4.**
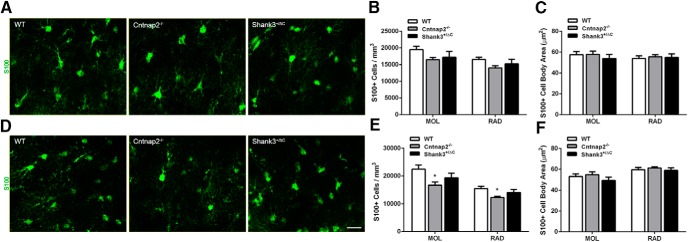
No consistent differences were observed in S100-labeled astrocyte density or cell body size across mouse autism models in the hippocampus. ***A***, ***D***, Representative images of sections immunolabeled with astrocyte marker S100 (green) from WT, Cntnap2^−/−^, and Shank3^+/ΔC^ mice in the dorsal (***A***) and ventral (***D***) MOL of the dentate gyrus. Scale bar, 20 µm, applies to all frames. ***B***, ***E***, Quantification of the density of S100-labeled astrocytes in the dorsal (***B***) and ventral (***E***) hippocampus. ***C***, ***F***, Quantification of S100 cell body area in the dorsal (***C***) and ventral (***F***) hippocampus. Error bars represent the SEM. *p < 0.05.

Examination of the astrocyte marker S100 revealed a slightly different pattern of staining. In the dorsal hippocampus, no significant differences were observed in S100^+^ cell density or cell body area between transgenic and wild-type mice in the MOL (density: *F*_(2,24)_ = 2.27, *p* = 0.13; cell body area: *F*_(2,24)_ = 0.39, *p* = 0.68) or in the RAD (density: *F*_(2,24)_ = 2.51, *p* = 0.10; cell body area: *F*_(2,24)_ = 0.11, *p* = 0.89; Fig. 4*A–C*). In the ventral hippocampal subregions, an overall significant difference in S100^+^ cell density was noted (MOL: *F*_(2,24)_ = 4.56, *p* = 0.02; RAD: *F*_(2,24)_ = 4.41, *p* = 0.02). *Post hoc* analyses revealed a significant decrease in the density of S100^+^ cells in both the MOL and RAD of Cntnap2^−/−^ mice compared with wild-type mice (MOL, *p* = 0.01; RAD, *p* = 0.01). However, no statistical difference was observed on this measure in the Shank3^+/ΔC^ mice (MOL, *p* = 0.31; RAD, *p* = 0.51; Fig. 4*D*,*E*). Furthermore, no statistical differences were observed in cell body area measurements in either the MOL (*F*_(2,24)_ = 0.92, *p* = 0.41) or RAD (*F*_(2,24)_ = 0.36, *p* = 0.70) of the ventral hippocampus (Fig. 4*F*).

### Microglia in the hippocampus

**Figure 5.**
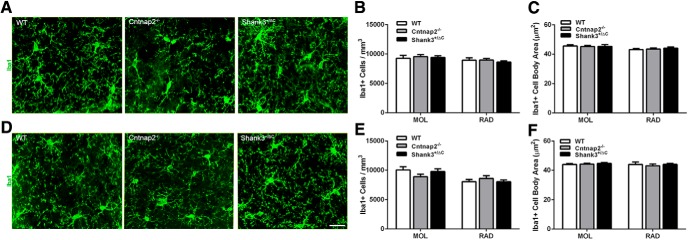
No differences were detected in Iba1-labeled microglia density and cell body size across mouse autism models in the hippocampus. ***A***, ***D***, Representative images of sections immunolabeled with microglia marker Iba1 (green) from WT, Cntnap2^−/−^, and Shank3^+/ΔC^ mice in the dorsal (***A***) and ventral (***D***) MOL of the dentate gyrus. Scale bar, 20 µm, applies to all frames. ***B***, ***E***, Quantification of the density of Iba1-labeled microglia in the dorsal (***B***) and ventral (***E***) hippocampus. ***C***, ***F***, Quantification of Iba1 cell body area in the dorsal (***C***) and ventral (***F***) hippocampus. Error bars represent the SEM.

Quantitative analyses of the number of Iba1^+^ microglial cells revealed no overall statistical differences in either the dorsal (MOL: *F*_(2,24)_ = 0.14, *p* = 0.87; RAD: *F*_(2,24)_ = 0.33, *p* = 0.72; Fig. 5*A*,*B*) or the ventral (MOL: *F*_(2,24)_ = 1.60, *p* = 0.22; RAD: *F*_(2,24)_ = 0.57, *p* = 0.57; Fig. 5*D*,*E*) hippocampus of transgenic mice compared with wild-type mice. Likewise, there was no consistent difference between groups in microglial cell body area (dorsal: MOL: *F*_(2,24)_ = 0.06, *p* = 0.94; SGZ: *F*_(2,24)_ = 0.69, *p* = 0.51; RAD: *F*_(2,24)_ = 0.39, *p* = 0.68; ventral: MOL: *F*_(2,24)_ = 0.26, *p* = 0.78; SGZ: *F*_(2,24)_ = 1.74, *p* = 0.20; RAD: H = 0.40, 2 df, *p* = 0.82; Fig. 5*C*,*F*) or the number of primary processes (dorsal: MOL: *F*_(2,24)_ = 1.35, *p* = 0.28; RAD: *F*_(2,24)_ = 1.16, *p* = 0.33; ventral: MOL: *F*_(2,24)_ = 3.13, *p* = 0.06; SGZ: *F*_(2,24)_ = 0.11, *p* = 0.90; RAD: *F*_(2,24)_ = 0.60, *p* = 0.56). While there was an overall significant difference in the number of primary processes of microglia in the dorsal SGZ (*F*_(2,24)_ = 4.12, *p* = 0.03), *post hoc* analyses showed that this effect was only in Shank3^+/ΔC^ mice compared with wild-type mice (WT vs Cntnap2^−/−^, *p* = 0.17; WT vs Shank3^+/ΔC^, *p* = 0.02).

**Figure 6.**
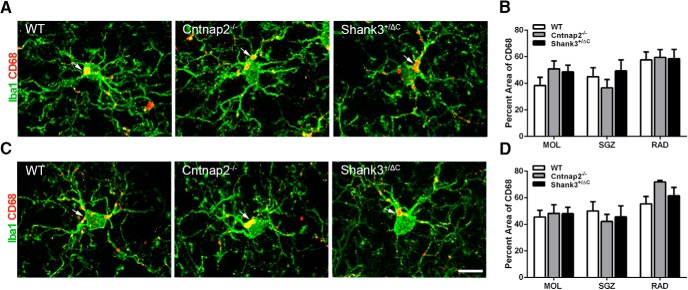
No differences were detected in the percentage area of CD68 in Iba1^+^ cells across mouse autism models in the hippocampus. ***A***, ***C***, Representative images of sections double labeled with microglia marker Iba1 (green) and microglial lysosomal marker CD68 (red) from WT, Cntnap2^−/−^, and Shank3^+/ΔC^ mice in the dorsal (***A***) and ventral (***C***) MOL of the dentate gyrus. Arrows point to CD68 aggregate labeling. Scale bar, 10 µm, applies to all frames. ***B***, ***D***, Quantification of the percentage area of CD68 in Iba1-labeled microglia cells in the dorsal (***B***) and ventral (***D***) hippocampus. Error bars represent the SEM.

Analysis of CD68 immunolabeling, an indicator of microglial reactivity, within Iba1^+^ microglia also yielded no statistically significant differences in the percentage of the labeled area among groups for the dorsal hippocampus (MOL: *F*_(2,24)_ = 1.36, *p* = 0.28; SGZ: *F*_(2,24)_ = 0.82, *p* = 0.45; RAD: *F*_(2,24)_ = 0.03, *p* = 0.98; Fig. 6*A*,*B*) and the ventral hippocampus (MOL: *F*_(2,24)_ = 0.07, *p* = 0.93; SGZ: *F*_(2,24)_ = 0.39, *p* = 0.68; RAD: H = 4.58, 2 df, *p* = 0.10; Fig. 6*C*,*D*). Furthermore, we found no difference in the number of CD68 aggregates in microglia in either the dorsal (MOL: *F*_(2,24)_ = 2.37, *p* = 0.12; SGZ: *F*_(2,24)_ = 2.78, *p* = 0.08; RAD: *F*_(2,24)_ = 0.52, *p* = 0.60) or ventral (MOL: *F*_(2,24)_ = 1.91, *p* = 0.17; SGZ: *F*_(2,24)_ = 2.62, *p* = 0.09; RAD: H = 2.40, 2 df, *p* = 0.30) hippocampus.

### Astroglia in the medial prefrontal cortex

**Figure 7.**
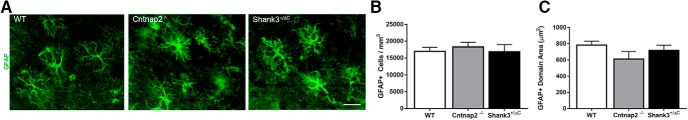
No differences in GFAP-labeled astrocyte density or domain size were detected in the mPFC. ***A***, Representative images of sections immunolabeled with GFAP (green) in the mPFC. Scale bar, 20 µm, applies to all frames. ***B***, Quantitative analysis of GFAP density in the mPFC. ***C***, Quantitative analysis of GFAP domain area in the mPFC. Error bars represent the SEM.

**Figure 8.**
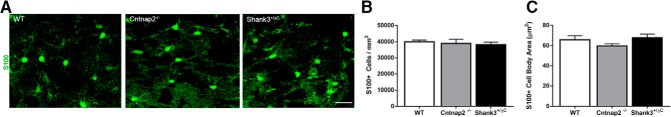
No differences in astrocyte number or cell body size were detected in the mPFC using the astrocytic marker S100. ***A***, Representative images of sections immunolabeled with S100 (green) in the mPFC. Scale bar, 20 µm, applies to all frames. ***B***, Quantitative analysis of S100 density in the mPFC. ***C***, Quantitative analysis of S100 cell body area in the mPFC. Error bars represent the SEM.

No significant differences were observed in GFAP^+^ cell density (*F*_(2,24)_ = 0.31, *p* = 0.74) or domain area (*F*_(2,24)_ = 1.56, *p* = 0.23) in the mPFC when comparing across genotype (Fig. 7). Similar to what was observed for GFAP-labeled tissue in the mPFC, quantitative analyses of S100^+^ density (*F*_(2,24)_ = 0.21, *p* = 0.81) and cell body area (*F*_(2,24)_ =1.61, *p* = 0.22) in the mPFC revealed no significant differences across genotypes (Fig. 8).

### Microglia in the medial prefrontal cortex

**Figure 9.**
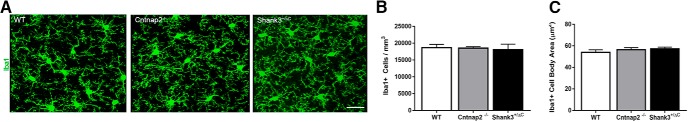
No differences in Iba1-labeled microglia density or cell body size were detected in the mPFC across groups. ***A***, Representative images of sections immunolabeled with Iba1 (green) in the mPFC. Scale bar, 20 µm, applies to all frames. ***B***, Quantitative analysis of Iba1 density in the mPFC. ***C***, Quantitative analysis of Iba1 cell body area in the mPFC. Error bars represent the SEM.

No significant differences in the density of Iba1^+^ microglia (*F*_(2,24)_ = 0.08, *p* = 0.92) and the cell body area of Iba1^+^ microglia (*F*_(2,24)_ =.64, *p* = 0.54) were observed across genotypes (Fig. 9). Likewise, no differences in the number of primary processes of Iba1^+^ microglia were detected across genotypes (*F*_(2,24)_ = 0.76, *p* = 0.48).

**Figure 10.**
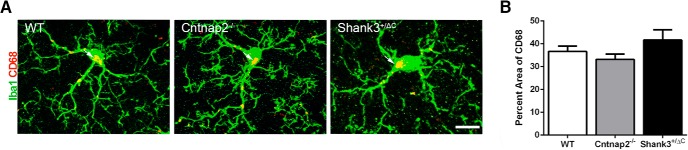
No differences were detected in the percentage area of CD68 in Iba1^+^ cells across mouse autism models in the mPFC. ***A***, Representative images of sections double labeled with microglia marker Iba1 (green) and microglial lysosomal marker CD68 (red) from WT, Cntnap2^−/−^, and Shank3^+/ΔC^ mice in the mPFC. Scale bar, 10 µm, applies to all frames. Arrows point to CD68 aggregate labeling. ***B***, Quantification of the percentage area of CD68 in Iba1-labeled microglia cells in the mPFC. Error bars represent the SEM.

No differences were observed in microglial activation, as measured by the percentage of the area of CD68 in Iba1^+^ cells (*F*_(2,24)_ = 1.93, *p* = 0.17; Fig. 10) as well as by counting the number of CD68 aggregates in Iba1^+^ cells (*F*_(2,24)_ = 1.00, *p* = 0.38) across genotypes.

### Astroglia in the striatum

**Figure 11.**
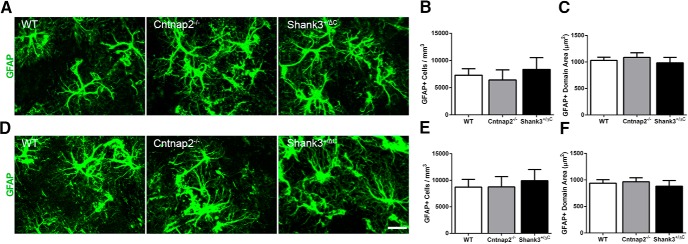
No differences were observed in GFAP-labeled astrocyte number and domain size across mouse autism models in the dorsal striatum. ***A***, ***D***, Representative images of sections immunolabeled with GFAP (green) from WT, Cntnap2^−/−^, and Shank3^+/ΔC^ mice in the DLS (***A***) and DMS (***D***). Scale bar, 20 µm, applies to all frames. ***B***, ***E***, Quantification of the density of GFAP-labeled astrocytes in the DLS (***B***) and DMS (***E***). ***C***, ***F***, Quantification of cell domain area of GFAP-labeled astrocytes in the DLS (***C***) and DMS (***F***). Error bars represent the SEM.

No significant differences were observed in the number of GFAP^+^ astrocytes between transgenic and wild-type mice in either the DLS (*F*_(2,24)_ = 0.39, *p* = 0.68; Fig. 11*A*,*B*) or in the DMS (*F*_(2,24)_ = 0.27, *p* = 0.77; Fig. 11*D*,*E*). Furthermore, no differences in the domain sizes of individual GFAP^+^ astrocytes were detected in the DLS (*F*_(2,24)_ = 0.38, *p* = 0.69; Fig. 11*C*) or in the DMS (*F*_(2,24)_ = 0.24, *p* = 0.79; Fig. 11*F*).

**Figure 12.**
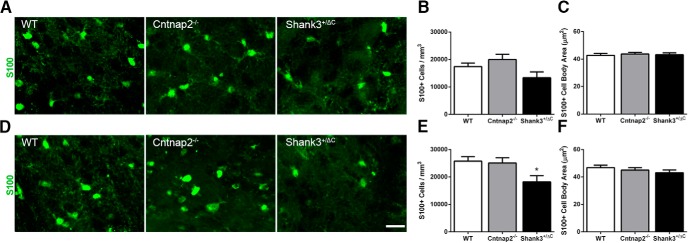
No consistent differences were observed in S100-labeled astrocyte number and cell body size across mouse autism models in the dorsal striatum. ***A***, ***D***, Representative images of sections immunolabeled with S100 (green) from WT, Cntnap2^−/−^, and Shank3^+/ΔC^ mice in the DLS (***A***) and DMS (***D***). Scale bar, 20 µm, applies to all frames. ***B***, ***E***, Quantification of the density of S100-labeled astrocytes in the DLS (***B***) and DMS (***E***). ***C***, ***F***, Quantification of cell body area of S100-labeled astrocytes in the DLS (***C***) and DMS (***F***). Error bars represent the SEM. *p < 0.05.

Similar to the analysis of GFAP^+^ astrocytes in the striatum, S100^+^ astrocytes showed no consistent differences between transgenic and wild-type mice in either the DLS (*F*_(2,24)_ = 3.20, *p* = 0.06; Fig. 12*A*,*B*) or in the DMS (*F*_(2,24)_ = 4.1, *p* = 0.03; Fig. 12*D*,*E*). Although an overall significant effect was observed for S100^+^ density in the DMS, *post hoc* comparisons revealed a significant decrease only in Shank3^+/ΔC^ mice compared with wild-type mice (WT vs Shank3^+/ΔC^, *p* = 0.03). This effect was not seen in Cntnap2^−/−^ mice (WT vs Cntnap2^−/−^, *p* > 0.99). Furthermore, no differences in S100^+^ cell body area were detected in the DLS (*F*_(2,24)_ = 0.16, *p* = 0.85; Fig. 12*C*) or in the DMS (*F*_(2,24)_ = 0.93, *p* = 0.41; Fig. 12*F*).

### Microglia in the striatum

**Figure 13.**
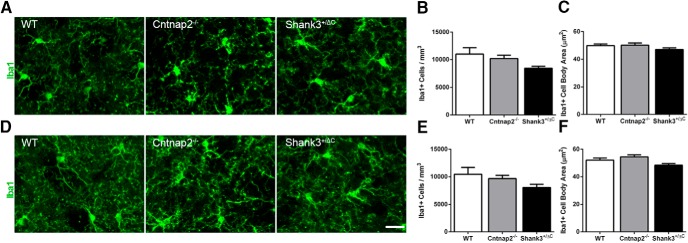
No consistent differences were observed in Iba1-labeled microglia number and cell body size across mouse models in the dorsal striatum. ***A***, ***D***, Representative images of sections immunolabeled with Iba1 (green) from WT, Cntnap2^−/−^, and Shank3^+/ΔC^ mice in the DLS (***A***) and DMS (***D***). Scale bar, 20 µm, applies to all frames. ***B***, ***E***, Quantification of the density of Iba1-labeled microglia in the DLS (***B***) and DMS (***E***). ***C***, ***F***, Quantification of the cell body area of Iba1-labeled microglia in the DLS (***C***) and DMS (***F***). Error bars represent the SEM.

There were no differences in the density of Iba1^+^ microglia among the two transgenic models and wild-type mice in either the DLS (H = 4.66, 2 df, *p* = 0.098; Fig. 13*A*,*B*) or the DMS (H = 3.16, 2 df, *p* = 0.21; Fig. 13*D*,*E*). Likewise, there was no overall statistical difference in microglial cell body area among transgenic models and wild-type mice in the DLS (*F*_(2,24)_ = 1.38, *p* = 0.27; Fig. 13*C*). Although an overall significant effect was observed for Iba1^+^ cell body area in the DMS (*F*_(2,24)_ = 3.76, *p* = 0.04; Fig. 13*F*), *post hoc* analysis revealed no significant differences between transgenic and wild-type mice (WT vs Shank3^+/ΔC^, *p* = 0.22; WT vs Cntnap2^−/−^, *p* = 0.49). No significant difference in the number of primary processes of Iba1^+^ microglia was detected in the DLS across genotype (*F*_(2,24)_ = 0.26, *p* = 0.77). Although an overall significant effect for the number of primary processes of Iba1^+^ microglia was observed in the DMS (*F*_(2,24)_ = 7.42, *p* = 0.0031), *post hoc* analysis revealed a significant decrease only in Cntnap2^−/−^ mice compared with wild-type mice (WT vs Cntnap2^−/−^, *p* = 0.0058). This effect was not seen in Shank3^+/ΔC^ mice (WT vs Shank3^+/ΔC^, *p* > 0.99).

**Figure 14.**
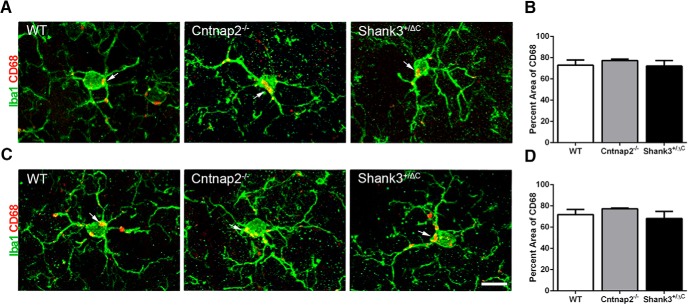
No differences were detected in the percentage area of CD68 in Iba1^+^ cells across mouse autism models in the dorsal striatum. ***A***, ***C***, Representative images of sections immunolabeled with Iba1 (green) and microglial lysosomal marker CD68 (red) from WT, Cntnap2^−/−^, and Shank3^+/ΔC^ mice in the DLS (***A***) and DMS (***C***). Arrows point to CD68 aggregate labeling. Scale bar, 10 µm, applies to all frames. ***B***, ***D***, Quantification of the percentage area of CD68 in Iba1-labeled microglia cells in DLS (***B***) and DMS (***D***). Error bars represent the SEM.

Iba1^+^ microglial cells were also analyzed for microglial activation through colabeling with CD68. Quantitative analysis of CD68^+^ percent area revealed no significant effect in the DLS (H = 0.32, 2 df, *p* = 0.85; Fig. 14*A*,*B*). Similarly, no significant difference in microglial activation was seen in the DMS (H = 0.75, 2 df, *p* = 0.69; Fig. 14*C*,*D*). Additionally, the number of CD68 aggregates in Iba1^+^ cells revealed no significant differences across genotype in either the DLS (H = 1.88, 2 df, *p* = 0.39) or the DMS (*F*_(2,24)_ = 0.89, *p* = 0.42).

### Differences in astroglia across brain regions

In our study, no consistent glial differences were found between wild-type and autism mouse models within the hippocampus, mPFC, or striatum other than in specialized astroglia in the SGZ known to serve as progenitor cells for immature neurons. Microscopic analyses of GFAP^+^ cells with astrocyte morphology revealed different profiles among the hippocampus, mPFC, and striatum (Figs. 2, 3, 7, 11). The density of GFAP-labeled cells (per cubic millimeter) in the dentate gyrus molecular layer and CA1 stratum radiatum of the hippocampus far exceeded that of both the mPFC and striatum. Furthermore, GFAP-labeled astrocytes in the mPFC were on average smaller in domain area compared with the hippocampus and striatum. The overall shape of the GFAP-labeled cells differed; astrocytes in the mPFC appeared to be more spherical in shape with more elaborate branched processes than those in the hippocampus (compare Figs. 3, 7). Similar differences in appearance were seen between the striatum and hippocampus, where astrocytes in the striatum appeared to have more branched processes in a spherical pattern, but were similar in domain area (compare Figs. 3, 11). Analyses from S100^+^ cells also revealed regional differences in cell density and size. The density of S100^+^ cells (per cubic millimeter) in the mPFC was approximately twice that of the DLS and dentate gyrus molecular layer and CA1 stratum radiatum of the hippocampus. Additionally, the average DLS cell body area of S100^+^ cells was smaller than all other regions (compare Figs. 4, 8, 12).

## Discussion

We searched for similar differences in markers of neuronal and glial plasticity in three brain regions in two genetic models of autism compared with wild-type controls. Of the measures we made, the only ones that exhibited similar differences across both ASD mouse models were those related to adult neurogenesis in the hippocampus, namely, the number of doublecortin-labeled cells and the number of radial glial-like progenitor cells. We observed no consistent changes in any other markers of glial plasticity, including the number or size of astrocytes in the hippocampus, mPFC, or striatum, and no differences in the number, size, or activation of microglia in the aforementioned brain regions across both genotypes. Despite not finding consistent differences in markers of astrocytes or microglia across genotypes, differences between genotypes unique to particular cell types were detected in both the hippocampus and striatum. In the hippocampus, GFAP^+^ cell domain areas were smaller in the dorsal stratum radiatum of Shank3^+/ΔC^ animals relative to wild-type controls. Similarly, fewer S100^+^ cells were found in Cntnap2^−/−^ animals in the dorsal molecular layer and stratum radiatum of the hippocampus. In the dorsomedial striatum, fewer S100^+^ cells were detected in Shank3^+/ΔC^ mice. Collectively, these findings suggest that shared differences in adult neurogenesis exist, but that potential differences in glial plasticity associated with ASD may be more subtle, and not necessarily general findings, in the adult brain.

We found a robust decrease in the number of new neurons, as well as in the number of radial glial-like progenitor cells, in the ventral, but not dorsal, dentate gyrus in both Cntnap2^−/−^ and Shank3^+/ΔC^ transgenic mice. The specificity of this finding to the ventral part of the hippocampus may be functionally relevant as, in the rodent, the ventral hippocampus has been linked to anxiety and stress regulation, whereas the dorsal hippocampus has been linked to spatial navigation learning and memory (Fanselow and Dong, 2010). Since ASD has high comorbidity with anxiety disorders (Simonoff et al., 2008; van Steensel et al., 2011), reduced numbers of new neurons may contribute to increased anxiety. Recent evidence suggests that, in addition to anxiety regulation, new neurons in the ventral dentate gyrus may mediate some social behaviors (Opendak et al., 2016). Our adult neurogenesis findings are in line with behavioral data showing that Cntnap2^−/−^ and Shank3^+/ΔC^ transgenic mice display more anxiety-like behaviors and deficits in social behaviors compared to wild-type mice (Peñagarikano et al., 2011; Duffney et al., 2015). Additionally, our data are also consistent with another study that showed that the BTBR mouse strain, which exhibits many behavioral deficits associated with ASD, has reduced the numbers of adult-born neurons in the hippocampus (Stephenson et al., 2011). However, this study did not differentiate between the dorsal and ventral portions of the dentate gyrus. To determine whether reduced numbers of immature neurons are a general phenomenon of ASD, future work should explore markers of adult neurogenesis in other mouse models, as well as in postmortem human ASD tissue.

Despite clear differences in the numbers of new neurons and astroglial progenitor cells in the ventral hippocampus, we detected no consistent differences across genotypes when looking at mature astrocytes or microglia in this brain region. These findings may be difficult to interpret given the heterogeneity of ASD. In particular, one problem with interpreting genotypic differences between mouse models of ASD is that the rare mutant variants account for <1% of all ASD cases (Devlin and Scherer, 2012). Comparing individuals with these specific abnormalities, similarities reflective of the core symptoms of ASD can be seen; however, differences in severity, seizure prevalence, muscle tone, developmental delay, and other comorbid conditions exist (Devlin and Scherer, 2012; Parikshak et al., 2013). While mouse models based on rare mutations offer useful models to study the development of ASD, there are often difficulties relating behavioral deficits in rodents to those seen in humans (Nestler and Hyman, 2010). These difficulties may be due, in part, to the likelihood that ASD is caused by multiple genetic factors, which together comprise an individual’s genetic liability (Geschwind, 2011). Nevertheless, the fact that many of the ASD candidate genes, including Cntnap2 and Shank3, are associated with both neuronal and glial plasticity as well as with synapse development, at least suggests the possibility of shared underlying mechanisms or biomarkers for many, if not all, forms of ASD (Cahoy et al., 2008; Peñagarikano et al., 2011; Jiang and Ehlers, 2013).

Postmortem studies show increased levels of glial markers in the brains of patients with ASD (Vargas et al., 2005; Morgan et al., 2010), and experimental studies have also linked glial dysregulation to ASD. Methyl-CpG-binding protein-2 (MECP2), a transcription factor that is expressed in neurons and glia, is atypically expressed in ASD and mutated in Rett syndrome, which produces many symptoms similar to those in ASD (Ballas et al., 2009; Maezawa et al., 2009). The absence of MECP2 in astrocytes impedes normal neuronal growth (Ballas et al., 2009). On the other hand, re-expression of MECP2 in astrocytes of MECP2-null mice restored these alterations in dendritic morphology and significantly improved Rett syndrome-like behaviors, such as locomotion and anxiety levels (Lioy et al., 2011). Another study (Maezawa and Jin, 2010) showed that knockout of MECP2 specifically in microglial cells causes stunted dendritic growth of hippocampal neurons and damages the postsynaptic elements of excitatory synapses by increasing the release of glutamate. Mice lacking Cx3Cr1, a chemokine receptor expressed by microglia that is involved in neuron–microglia cross talk, not only have reduced numbers of new neurons (Bachstetter et al., 2011), but they also have an autistic-like behavioral phenotype, including impaired social interaction and increased repetitive behaviors (Zhan et al., 2014). These transgenic mice also exhibit alterations in neural circuitry commonly associated with ASD, including impaired synaptic pruning, weak synaptic transmission, and decreased long-range functional connectivity (Paolicelli et al., 2011; Zhan et al., 2014).

Although we did not find common abnormalities in astrocytes or microglia across the two autism mouse models, our findings do not exclude the possibility that astrocytic and microglial dysfunction are important in the etiology of ASD. Indeed, abnormalities in these cell types may have existed early in life that had resolved by adulthood. The mechanisms of ASD are known to have their onset in the first year of human postnatal life, and many ASD genes are coexpressed in mid-gestation in the developing human brain (Willsey et al., 2013). In mice, the mechanisms of neocortical synapse proliferation and elimination are likely to reach a peak in the first 2 months of postnatal life (Alvarez and Sabatini, 2007). Our measurements, which were performed on mice at later ages, would tend to emphasize aberrations in plasticity mechanisms that persisted even after this period has ended. In this regard, it is notable that our positive results were found in the dentate gyrus, a structure that undergoes a considerable amount of neurogenesis and other forms of plasticity throughout adult life. One potential mechanism is that proper synapse formation and elimination does not occur in ASD individuals, producing abnormal neural connectivity. CD68, a microglial lysosomal marker associated with the engulfment of synapses, is present in high quantities in the mouse brain early in development compared with adulthood (Schafer et al., 2012). Since both astrocytes and microglia play a role in shaping neural circuitry by actively engulfing weak synapses during early life (Paolicelli et al., 2011; Schafer et al., 2012; Chung et al., 2013), glial dysfunction leading to ASD may occur at an early age. In our study, we examined 5- to 6-month-old male mice. The adult time point was specifically chosen because most reported autism-like behaviors for these transgenic lines were examined in adult mice (Peñagarikano et al., 2011; Duffney et al., 2015). Thus, future work is needed to determine whether a more consistent and robust alteration in glial cells occurs in mouse models of ASD at younger ages.

It is also possible that astrocyte and microglia abnormalities exist, but are not detectable using our specific measures. Although Iba1 is a well known marker of microglia and robustly labels all cells of macrophage origin (Ito et al., 1998), heterogeneity in astrocytic cell populations is an important consideration. Despite the fact that both GFAP and S100 are common markers for astrocytes, these markers only label a relatively small proportion of astrocytes (Cahoy et al., 2008). In fact, not all astrocytes colabel with GFAP and S100 (Cahoy et al., 2008; Brockett et al., 2015), suggesting that there are different subpopulations of astrocytes, which may be differentially altered in ASD. Additionally, GFAP does not label the entirety of astrocytic processes and is largely absent from astrocytic end feet (Bushong et al., 2002), so morphology could be altered in ASD but in regions of the cell that do not label with GFAP. Both microglia and astrocytes are highly dynamic and can change morphologies within a matter of minutes (Nimmerjahn et al., 2005; Theodosis et al., 2008; Perez-Alvarez et al., 2014). Our findings leave open the possibility that more subtle changes in morphology may occur in ASD as well as in other brain regions that are not explored here but are implicated in ASD (Schumann and Amaral, 2006; Dichter et al., 2012; Wang et al., 2014).

Our findings, along with those of a previous study (Stephenson et al., 2011), suggest that diminished adult neurogenesis in the hippocampus may be a feature shared by many ASD mouse models. Our additional finding of reduced numbers of astroglial cells with radial morphology in the ventral SGZ suggest that decreased numbers of immature neurons occur as a result of fewer progenitor cells. Since radial glial cells serve as neuronal progenitors during development as well as in adulthood, it is possible that these findings reflect residual evidence of a neurogenesis deficit that exists during development in multiple brain regions. Indeed, studies suggest that some of the gross structural abnormalities detected in the postmortem ASD brain may arise from aberrant neurogenesis (Williams and Casanova, 2010). However, the extent to which deficits in radial glial progenitor cells and neurogenesis contribute to symptoms of ASD remains to be determined.

## Synthesis

Reviewing Editor: Liisa Galea, University of British Columbia. Decisions are customarily a result of the Reviewing Editor and the peer reviewers coming together and discussing their recommendations until a consensus is reached. When revisions are invited, a fact-based synthesis statement explaining their decision and outlining what is needed to prepare a revision will be listed below. The following reviewer(s) agreed to reveal their identity: Jason Snyder. For some manuscripts transferred from JNeurosci, a decision is made to accept the manuscript without peer review and a brief statement to this effect will follow.

This is a well-written, comprehensive paper that reports quantitative assays of neurogenesis and glial cell numbers in PFC, hippocampus and striatum of two mouse models of Autism-spectrum disorders (ASDs), Cntnap2-/- and Shank3+/ΔC transgenic mice. The rationale for the experiments is that postmortem studies suggest that neurogenesis and glial populations are abnormal in ASD, but it is not known whether these defects are present across relevant mouse models. The main results are that both ASD mouse models displayed a decrease in the number of immature neurons and radial glial progenitor cells in the ventral dentate gyrus, but no consistent differences were detected in the number or size of astrocytes or microglia in any other brain regions examined. The latter result suggests that glial abnormalities are not necessary for ASD behavioral phenotypes. The cell quantifications are thorough, particularly with respect to glial populations, and will be useful for other researchers interested in ASDs. In addition, the parallel reductions in neurogenesis and radial glia number in both mouse models suggests that these phenotypes deserve more attention.

1. The reduction in newborn neurons and radial glia calls out for a more mechanistic analysis of changes in the relative proportion of maturation stages. This is one place where a more "mechanistic" analysis could have been incorporated without much extra work. One way to begin to answer this question would be to do an analysis of the phenotype of the DCX-expressing neurons. In this way you could address whether you see proportionally different amounts of Type I, type II or type II DCX-expressing neurons between the different mouse lines?

2. More detail is requested in the Methods section:

1)stereological quantification. Were cells counted exhaustively or was an estimator used, such as optical fractionator? Were guard zones used to avoid overcounting? The use/nonuse of these methods would probably not affect the conclusions of this study but could affect the overall density values and thus will be relevant to other researchers interested in the cell counts.

2)How were the z-stacks obtained for the cross-sectional measurements? Microscope, objective, imaging parameters.

3)Which portions of CA1, dorsal-medial striatum, mPFC were examined (beyond stating the "center region")? (CA1 isn't even mentioned in the methods) The entire region rostro-caudally, medio-laterally, dorsoventrally? All neocortical layers? What were the dorsoventral boundaries for the dentate gyrus analyses?

4)For the cell area measurements it must be subregional analyses since only 10-50 cells were analysed - how were these chosen/sampled?

5)Is there functionally significant difference between punctate and aggregate CD68 staining? - perhaps a description of the significance could be included and the reason behind measuring them differently.

3. CD68 is a microglia-specific lysosome marker relevant to phagocytic activity. The Shaffer paper defines activation by taking into account both CD68 expression and microglia morphology using a 0-5 scale, however in this manuscript only CD68 expression but not morphology of Iba-1 cells are analyzed together. If so this is slightly different from the Shaffer method and should be acknowledged ( and perhaps important to also examined according to the morphology of the processes from Iba-1 expressing cells). It is important to acknowledge that there is more to microglial activation than phagocytosis, and by limiting activation analysis to CD68 expression and ignoring morphology some important information may be lost.

4. For the punctate and aggregate CD68 staining it appears as though these are just different sized chunks of CD68 protein. Differentially weighting anything larger than 3um, by multiplying it 10x more than staining chunks smaller than 3um seems a bit crude. For example, a CD68 signal that is 3.5um would be multiplied 10x more than a signal that measures only 2.5um across. And then are these differentially weighted counts combined? I assume so. Binning them into quartiles will also lose quantitative information. Why not go back to these images and take the CD68 channel and measure the total area occupied by the CD68 signal? This seems like it would be more accurate and would introduce fewer systematic quantitative biases.

5. The Results section is quite long and could be condensed without reducing the important information but analysis type could be reconsidered- for example instead of conducting two one-way ANOVAs separately for each area of investigation ( e.g. dorsal versus ventral) you could condense these two analyses into one repeated measures ANOVA with dorsal/ventral as your within factor. The same is true of the analyses of the stratum radiatum versus molecular layer ( instead of 2 one-way ANOVAs, 1 RM ANOVA could be conducted). However the effects seen are quite large and thus likely your outcome will not be affected and we leave it up to the authors whether or not they wish to include area as a within-subjects factor in their analyses.

6. Table 1 is very much appreciated. In addition to normality ANOVA is sensitive to large variations in variance, thus it would be better to have HOV information in addition to normality within this Table ( or a statement indicating both assumptions are tenable for each analysis).

7. In the introduction, in the second and third sentence, although it is obvious make it clear that these are post-mortem findings in individuals with ASD (only in boys/men?).

8. Please add "male" to the last sentence in Introduction to make it clear this is only in males.

9. And finally a question for interest may also be whether the relative proportion of new glia and new neurons is altered with each strain? Given evidence that glial populations are affected in ASDs, there may well be differences in adult gliogenesis. Is the mode of division of radial glia altered? Is their rate of division changed? Is neuronal proliferation or survival altered? These are questions that are given as food for thought rather than something that needs to be addressed in the manuscript itself.

## References

[B1] Alvarez VA, Sabatini B (2007) Anatomical and physiological plasticity of dendritic spines. Annu Rev Neurosci 30:79–97. 10.1146/annurev.neuro.30.051606.094222 17280523

[B2] American Psychiatric Association (2013) Diagnostic and statistical manual of mental disorders: DSM-5. Washington, DC: American Psychiatric Association.

[B3] Ashwood P, Krakowiak P, Hertz-Picciotto I, Hansen R, Pessah I, Van de Water J (2011) Elevated plasma cytokines in autism spectrum disorders provide evidence of immune dysfunction and are associated with impaired behavioral outcome. Brain Behav Immun 25:40–45. 10.1016/j.bbi.2010.08.003 20705131 PMC2991432

[B4] Atladóttir HO, Thorsen P, Østergaard L, Schendel DE, Lemcke S, Abdallah M, Parner ET (2010) Maternal infection requiring hospitalization during pregnancy and autism spectrum disorders. J Autism Dev Disord 40:1423–1430. 10.1007/s10803-010-1006-y 20414802

[B5] Bachstetter AD, Morganti JM, Jernberg J, Schlunk A, Mitchell SH, Brewster KW, Hudson CE, Cole MJ, Harrison JK, Bickford PC, Gemma C (2011) Fractalkine and CX_3_CR1 regulate hippocampal neurogenesis in adult and aged rats. Neurobiol Aging 32:2030–2044. 10.1016/j.neurobiolaging.2009.11.022 20018408 PMC2889032

[B6] Ballas N, Lioy DT, Grunseich C, Mandel G (2009) Non-cell autonomous influence of MeCP2-deficient glia on neuronal dendritic morphology. Nat Neurosci 12:311–317. 10.1038/nn.2275 19234456 PMC3134296

[B7] Brockett AT, LaMarca EA, Gould E (2015) Physical exercise enhances cognitive flexibility as well as astrocytic and synaptic markers in the medial prefrontal cortex. PLoS One 10:e0124859. 10.1371/journal.pone.0124859 25938418 PMC4418599

[B8] Bushong EA, Martone ME, Jones YZ, Ellisman MH (2002) Protoplasmic astrocytes in CA1 stratum radiatum occupy separate anatomical domains. J Neurosci 22:183–192. 11756501 10.1523/JNEUROSCI.22-01-00183.2002 PMC6757596

[B9] Cahoy JD, Emery B, Kaushal A, Foo LC, Zamanian JL, Christopherson KS, Xing Y, Lubischer JL, Krieg PA, Krupenko SA, Thompson WJ, Barres BA (2008) A transcriptome database for astrocytes, neurons and oligodendrocytes: a new resource for understanding brain development and function. J Neurosci 28:264–278. 10.1523/JNEUROSCI.4178-07.2008 18171944 PMC6671143

[B10] Carper RA, Courchesne E (2005) Localized enlargement of the frontal cortex in early autism. Biol Psychiatry 57:126–133. 10.1016/j.biopsych.2004.11.005 15652870

[B11] Christensen DL, Baio J, Braun KV, Bilder D, Charles J, Constantino JN, Daniels J, Durkin MS, Fitzgerald RT, Kurzius-Spencer M, Lee LC, Pettygrove S, Robinson C, Schulz E, Wells C, Wingate MS, Zahorodny W, Yeargin-Allsopp M (2012) Prevalence and characteristics of autism spectrum disorder among children aged 8 years — autism and developmental disabilities monitoring network, 11 sites, United States, 2012. MMWR Surveill Summ 65:1–23. 10.15585/mmwr.ss6513a1 PMC6237390 30439868

[B12] Christopherson KS, Ullian EM, Stokes CC, Mullowney CE, Hell JW, Agah A, Lawler J, Mosher DF, Bornstein P, Barres BA (2005) Thrombospondins are astrocyte-secreted proteins that promote CNS synaptogenesis. Cell 120:421–433. 10.1016/j.cell.2004.12.020 15707899

[B13] Chung WS, Clarke LE, Wang GX, Stafford BK, Sher A, Chakraborty C, Joung J, Foo LC, Thompson A, Chen C, Smith SJ, Barres BA (2013) Astrocytes mediate synapse elimination through MEGF10 and MERTK pathways. Nature 504:394–400. 10.1038/nature12776 24270812 PMC3969024

[B14] Devlin B, Scherer SW (2012) Genetic architecture in autism spectrum disorder. Curr Opin Genet Dev 22:229–237. 10.1016/j.gde.2012.03.002 22463983

[B15] Dichter GS, Felder JN, Green SR, Rittenberg AM, Sasson NJ, Bodfish JW (2012) Reward circuitry function in autism spectrum disorders. Soc Cogn Affect Neurosci 7:160–172. 10.1093/scan/nsq095 21148176 PMC3277365

[B16] Duffney LJ, Zhong P, Wei J, Matas E, Cheng J, Qin L, Ma K, Dietz DM, Kajiwara Y, Buxbaum JD, Yan Z (2015) Autism-like deficits in Shank3-deficient mice are rescued by targeting actin regulators. Cell Rep 11:1400–1413. 10.1016/j.celrep.2015.04.064 26027926 PMC4464902

[B17] Fanselow MS, Dong HW (2010) Are the dorsal and ventral hippocampus functionally distinct structures? Neuron 65:7–19. 10.1016/j.neuron.2009.11.031 20152109 PMC2822727

[B18] Franklin KBJ, Paxinos G (2008) The mouse brain in stereotaxic coordinates, Ed 3. New York: Elsevier Science.

[B19] Geschwind DH (2011) Genetics of autism spectrum disorder. Trends Cogn Sci 15:409–416. 10.1016/j.tics.2011.07.003 21855394 PMC3691066

[B20] Gdalyahu A, Lazaro M, Penagarikano O, Golshani P, Trachtenberg JT, Geschwind DH (2015) The autism related protein contactin-associated protein-like 2 (CNTNAP2) stabilizes new spines: an in vivo mouse study. PLoS One 10:e0125633 10.1371/journal.pone.0125633 25951243 PMC4423902

[B21] Goh S, Peterson BS (2012) Imaging evidence for disturbances in multiple learning and memory systems in persons with autism spectrum disorders. Dev Med Child Neurol 54:208–213. 10.1111/j.1469-8749.2011.04153.x 22269006

[B22] Henneberger C, Papouin T, Oliet SH, Rusakov DA (2010) Long-term potentiation depends on release of D-serine from astrocytes. Nature 463:232–236. 10.1038/nature08673 20075918 PMC2807667

[B23] Hutsler JJ, Zhang H (2010) Increased dendritic spine densities on cortical projection neurons in autism spectrum disorders. Brain Res 1309:83–94. 10.1016/j.brainres.2009.09.120 19896929

[B24] Ito D, Imai Y, Ohsawa K, Nakajima K, Fukuuchi Y, Kohsaka S (1998) Microglia-specific localisation of a novel calcium binding protein, Iba1. Brain Res Mol Brain Res 57:1–9. 9630473 10.1016/s0169-328x(98)00040-0

[B25] Jiang YH, Ehlers MD (2013) Modeling autism by SHANK gene mutations in mice. Neuron 78:8–27. 10.1016/j.neuron.2013.03.016 23583105 PMC3659167

[B26] Kinney DK, Munir KM, Crowley DJ, Miller AM (2008) Prenatal stress and risk for autism. Neurosci Biobehav Rev 32:1519–1532. 10.1016/j.neubiorev.2008.06.004 18598714 PMC2632594

[B27] Kondo S, Kohsaka S, Okabe S (2011) Long-term changes of spine dynamics and microglia after transient peripheral immune response triggered by LPS in vivo. Mol Brain 4:27 10.1186/1756-6606-4-27 21682853 PMC3138393

[B28] Kouser M, Speed HE, Dewey CM, Reimers JM, Widman AJ, Gupta N, Liu S, Jaramillo TC, Bangash M, Xiao B, Worley PF, Powell CM (2013) Loss of predominant Shank3 isoforms results in hippocampus-dependent impairments in behavior and synaptic transmission. J Neurosci 33:18448–18468. 10.1523/JNEUROSCI.3017-13.2013 24259569 PMC3834052

[B29] Kucukdereli H, Allen NJ, Lee AT, Feng A, Ozlu MI, Conatser LM, Chakraborty C, Workman G, Weaver M, Sage EH, Barres BA, Eroglu C (2011) Control of excitatory CNS synaptogenesis by astrocyte-secreted proteins Hevin and SPARC. Proc Natl Acad Sci U S A 108:E440–E449. 10.1073/pnas.1104977108 21788491 PMC3156217

[B30] Langen M, Schnack HG, Nederveen H, Bos D, Lahuis BE, de Jonge MV, van Engeland H, Durston S (2009) Changes in the development trajectories of striatum in autism. Biol Psychiatry 66:327–333. 10.1016/j.biopsych.2009.03.017 19423078

[B31] Lázaro MT, Golshani P (2015) The utility of rodent models of autism spectrum disorder. Curr Opin Neurol 28:103–109. 10.1097/WCO.0000000000000183 25734952 PMC4476903

[B32] Lioy DT, Garg SK, Monaghan CE, Raber J, Foust KD, Kaspar BK, Hirrlinger PG, Kirchhoff F, Bissonnette JM, Ballas N, Mandel G (2011) A role for glia in the progression of Rett's syndrome. Nature 475:497–500. 10.1038/nature10214 21716289 PMC3268776

[B33] Lugert S, Basak O, Knuckles P, Haussler U, Fabel K, Götz M, Haas CA, Kempermann G, Taylor V, Giachino C (2010) Quiescent and active hippocampal neural stem cells with distinct morphologies respond selectively to physiological and pathological stimuli and aging. Cell Stem Cell 6:445–456. 10.1016/j.stem.2010.03.017 20452319

[B34] Maezawa I, Jin LW (2010) Rett syndrome microglia damage dendrites and synapses by the elevated release of glutamate. J Neurosci 30:5346–5356. 10.1523/JNEUROSCI.5966-09.2010 20392956 PMC5533099

[B35] Maezawa I, Swanberg S, Harvey D, LaSalle JM, Jin LW (2009) Rett syndrome astrocytes are abnormal and spread MeCP2 deficiency through gap junctions. J Neurosci 29:5051–5061. 10.1523/JNEUROSCI.0324-09.2009 19386901 PMC3436907

[B36] Morgan JT, Chana G, Pardo CA, Achim C, Semendeferi K, Buckwalter J, Courchesne E, Everall IP (2010) Microglial activation and increased microglial density observed in the dorsolateral prefrontal cortex in autism. Biol Psychiatry 68:368–376. 10.1016/j.biopsych.2010.05.024 20674603

[B37] Nestler EJ, Hyman SE (2010) Animal models of neuropsychiatric disorders. Nat Neurosci 13:1161–1169. 10.1038/nn.2647 20877280 PMC3750731

[B38] Nimmerjahn A, Kirchhoff F, Helmchen F (2005) Resting microglial cells are highly dynamic surveillants of brain parenchyma in vivo. Science 308:1314–1318. 10.1126/science.1110647 15831717

[B39] Oberheim NA, Takano T, Han X, He W, Lin JH, Wang F, Xu Q, Wyatt JD, Pilcher W, Ojemann JG, Ransom BR, Goldman SA, Nedergaard M (2009) Uniquely hominid features of adult human astrocytes. J Neurosci 29:3276–3287. 10.1523/JNEUROSCI.4707-08.2009 19279265 PMC2819812

[B40] Opendak M, Gould E (2015) Adult neurogenesis: a substrate for experience-dependent change. Trends Cogn Sci 19:151–161. 10.1016/j.tics.2015.01.001 25715908

[B41] Opendak M, Offit L, Monari P, Schoenfeld T, Sonti A, Cameron HA, Gould E (2016) Lasting adaptations in social behavior produced by social disruption and inhibition of adult neurogenesis. J Neurosci 36:7027–7038. 10.1523/JNEUROSCI.4435-15.2016 27358459 PMC4926244

[B42] Ozonoff S, Young GS, Carter A, Messinger D, Yirmiya N, Zwaigenbaum L, Bryson S, Carver LJ, Constantino JN, Dobkins K, Hutman T, Iverson JM, Landa R, Rogers SJ, Sigman M, Stone WL (2011) Recurrence risk for autism spectrum disorders: a baby siblings research consortium study. Pediatrics 128:e488–e495. 10.1542/peds.2010-2825 21844053 PMC3164092

[B43] Parikshak NN, Luo R, Zhang A, Won H, Lowe JK, Chandran V, Horvath S, Geschwind DH (2013) Integrative functional genomic analyses implicate specific molecular pathways and circuits in autism. Cell 155:1008–1021. 10.1016/j.cell.2013.10.031 24267887 PMC3934107

[B44] Paolicelli RC, Bolasco G, Pagani F, Maggi L, Scianni M, Panzanelli P, Giustetto M, Ferreira TA, Guiducci E, Dumas L, Ragozzino D, Gross CT (2011) Synaptic pruning by microglia is necessary for normal brain development. Science 333:1456–1458. 10.1126/science.1202529 21778362

[B45] Peñagarikano O, Abrahams BS, Herman EI, Winden KD, Gdalyahu A, Dong H, Sonnenblick LI, Gruver R, Almajano J, Bragin A, Golshani P, Trachtenberg JT, Peles E, Geschwind DH (2011) Absence of CNTNAP2 leads to epilepsy, neuronal migration abnormalities, and core autism-related deficits. Cell 147:235–246. 10.1016/j.cell.2011.08.040 21962519 PMC3390029

[B46] Penzes P, Cahill ME, Jones KA, VanLeeuwen JE, Woolfrey KM (2011) Dendritic spine pathology in neuropsychiatric disorders. Nat Neurosci 14:285–293. 10.1038/nn.2741 21346746 PMC3530413

[B47] Perez-Alvarez A, Navarrete M, Covelo A, Martin ED, Araque A (2014) Structural and functional plasticity of astrocyte processes and dendritic spine interactions. J Neurosci 34:12738–12744. 10.1523/JNEUROSCI.2401-14.2014 25232111 PMC6705321

[B48] Rosenberg RE, Law JK, Yenokyan G, McGready J, Kaufmann WE, Law PA (2009) Characteristics and concordance of autism spectrum disorders among 277 twin pairs. Arch Pediatr Adolesc Med 163:907–914. 10.1001/archpediatrics.2009.98 19805709

[B49] Russell AJ, Murphy CM, Wilson E, Gillan N, Brown C, Robertson DM, Craig MC, Deeley Q, Zinkstok J, Johnston K, McAlonan GM, Spain D, Murphy DG (2016) The mental health of individuals referred for assessment of autism spectrum disorder in adulthood: a clinic report. Autism 20:623–627. 10.1177/1362361315604271 26471427

[B50] Schafer DP, Lehrman EK, Kautzman AG, Koyama R, Mardinly AR, Yamasaki R, Ransohoff RM, Greenberg ME, Barres BA, Stevens B (2012) Microglia sculpt postnatal neural circuits in an activity and complement-dependent manner. Neuron 74:691–705. 10.1016/j.neuron.2012.03.026 22632727 PMC3528177

[B51] Schumann CM, Amaral DG (2006) Stereological analysis of amygdala neuron number in autism. J Neurosci 26:7674–7679. 10.1523/JNEUROSCI.1285-06.2006 16855095 PMC6674270

[B52] Schumann CM, Hamstra J, Goodlin-Jones BL, Lotspeich LJ, Kwon H, Buonocore MH, Lammers CR, Reiss AL, Amaral DG (2004) The amygdala is enlarged in children but not adolescents with autism; the hippocampus is enlarged at all ages. J Neurosci 24:6392–6401. 10.1523/JNEUROSCI.1297-04.2004 15254095 PMC6729537

[B53] Simonoff E, Pickles A, Charman T, Chandler S, Loucas T, Baird G (2008) Psychiatric disorders in children with autism spectrum disorders: prevalence, comorbidity, and associated factors in a population-derived sample. J Am Acad Child Adolesc Psychiatry 47:921–929. 10.1097/CHI.0b013e318179964f 18645422

[B54] Stephenson DT, O'Neill SM, Narayan S, Tiwari A, Arnold E, Samaroo HD, Du F, Ring RH, Campbell B, Pletcher M, Vaidya VA, Morton D (2011) Histopathologic characterization of the BTBR mouse model of autistic-like behavior reveals selective changes in neurodevelopmental proteins and adult hippocampal neurogenesis. Mol Autism 2:7. 10.1186/2040-2392-2-7 21575186 PMC3135520

[B55] Theodosis DT, Poulain DA, Oliet SH (2008) Activity-dependent structural and functional plasticity of astrocyte-neuron interactions. Physiol Rev 88:983–1008. 10.1152/physrev.00036.2007 18626065

[B56] van Steensel FJ, Bögels SM, Perrin S (2011) Anxiety disorders in children and adolescents with autistic spectrum disorders: a meta-analysis. Clin Child Fam Psychol Rev 14:302–317. 10.1007/s10567-011-0097-0 21735077 PMC3162631

[B57] Vargas DL, Nascimbene C, Krishnan C, Zimmerman AW, Pardo C (2005) Neuroglial activation and neuroinflammation in the brain of patients with autism. Ann Neurol 57:67–81. 10.1002/ana.20315 15546155

[B58] Vasa RA, Mazurek MO (2015) An update on anxiety in youth with autism spectrum disorders. Curr Opin Psychiatry 28:83–90. 10.1097/YCO.0000000000000133 25602249 PMC5764108

[B59] Wang SS-H, Kloth AD, Badura A (2014) The cerebellum, sensitive periods, and autism. Neuron 83:518–532. 10.1016/j.neuron.2014.07.016 25102558 PMC4135479

[B60] Williams EL, Casanova MF (2010) Autism or autisms? Finding the lowest common denominator. Bol Asoc Med P R 102:17–24. 21766543

[B61] Willsey AJ, Sanders SJ, Li M, Dong S, Tebbenkamp AT, Muhle RA, Reilly SK, Lin L, Fertuzinhos S, Miller JA, Murtha MT, Bichsel C, Niu W, Cotney J, Ercan-Sencicek AG, Gockley J, Gupta AR, Han W, He X, Hoffman EJ,. (2013) Coexpression networks implicate human midfetal deep cortical projection neurons in the pathogenesis of autism. Cell 155:997–1007. 10.1016/j.cell.2013.10.020 24267886 PMC3995413

[B62] Xu N, Li X, Zhong Y (2015) Inflammatory cytokines: potential biomarkers of immunologic dysfunction in autism spectrum disorders. Mediators Inflamm 2015:531518. 10.1155/2015/531518 25729218 PMC4333561

[B63] Zager A, Peron JP, Mennecier G, Rodrigues SC, Aloia TP, Palermo-Neto J (2015) Maternal immune activation in late gestation increases neuroinflammation and aggravates experimental autoimmune encephalomyelitis in the offspring. Brain Behav Immun 43:159–171. 10.1016/j.bbi.2014.07.021 25108214

[B64] Zhan Y, Paolicelli RC, Sforazzini F, Weinhard L, Bolasco G, Pagani F, Vyssotski AL, Bifone A, Gozzi A, Ragozzino D, Gross CT (2014) Deficient neuron-microglia signaling results in impaired functional brain connectivity and social behavior. Nat Neurosci 17:400–406. 10.1038/nn.3641 24487234

